# Chemokines Modulate Immune Surveillance in Tumorigenesis, Metastasis, and Response to Immunotherapy

**DOI:** 10.3389/fimmu.2019.00333

**Published:** 2019-02-27

**Authors:** Anna E. Vilgelm, Ann Richmond

**Affiliations:** ^1^Tennessee Valley Healthcare System, Department of Veterans Affairs, Nashville, TN, United States; ^2^Department of Pharmacology, Vanderbilt University School of Medicine, Nashville, TN, United States

**Keywords:** chemokine, cancer, immune surveillance, immune therapy, metastasis, chemokine receptor

## Abstract

Chemokines are small secreted proteins that orchestrate migration and positioning of immune cells within the tissues. Chemokines are essential for the function of the immune system. Accumulating evidence suggest that chemokines play important roles in tumor microenvironment. In this review we discuss an association of chemokine expression and activity within the tumor microenvironment with cancer outcome. We summarize regulation of immune cell recruitment into the tumor by chemokine-chemokine receptor interactions and describe evidence implicating chemokines in promotion of the “inflamed” immune-cell enriched tumor microenvironment. We review both tumor-promoting function of chemokines, such as regulation of tumor metastasis, and beneficial chemokine roles, including stimulation of anti-tumor immunity and response to immunotherapy. Finally, we discuss the therapeutic strategies target tumor-promoting chemokines or induce/deliver beneficial chemokines within the tumor focusing on pre-clinical studies and clinical trials going forward. The goal of this review is to provide insight into comprehensive role of chemokines and their receptors in tumor pathobiology and treatment.

## Introduction

Migration of the immune cells to specific organs is controlled in part by small proteins called chemokines (i.e., chemotactic cytokines) (1, 2). Chemokines bind to seven transmembrane G protein-coupled receptors that trigger intracellular signaling that drives cell polarization, adhesion, and migration (3, 4). They are divided into four families based upon structure: CXC, CC, CX3C, and C chemokines. The receptors follow a similar nomenclature system, based upon the family of chemokines to which they bind. In addition there is a family of atypical chemokine receptors that do not directly couple to G proteins, but are reported to have a variety of roles in development, homeostasis, inflammatory disease, infection, and cancer (5). Chemokines are also classified as homeostatic or inflammatory (4, 6–8) and both subsets play important roles in cancer (9, 10).

## Chemokine/Chemokine Receptors in the Regulation of Leukocytes

**Figure 1:**
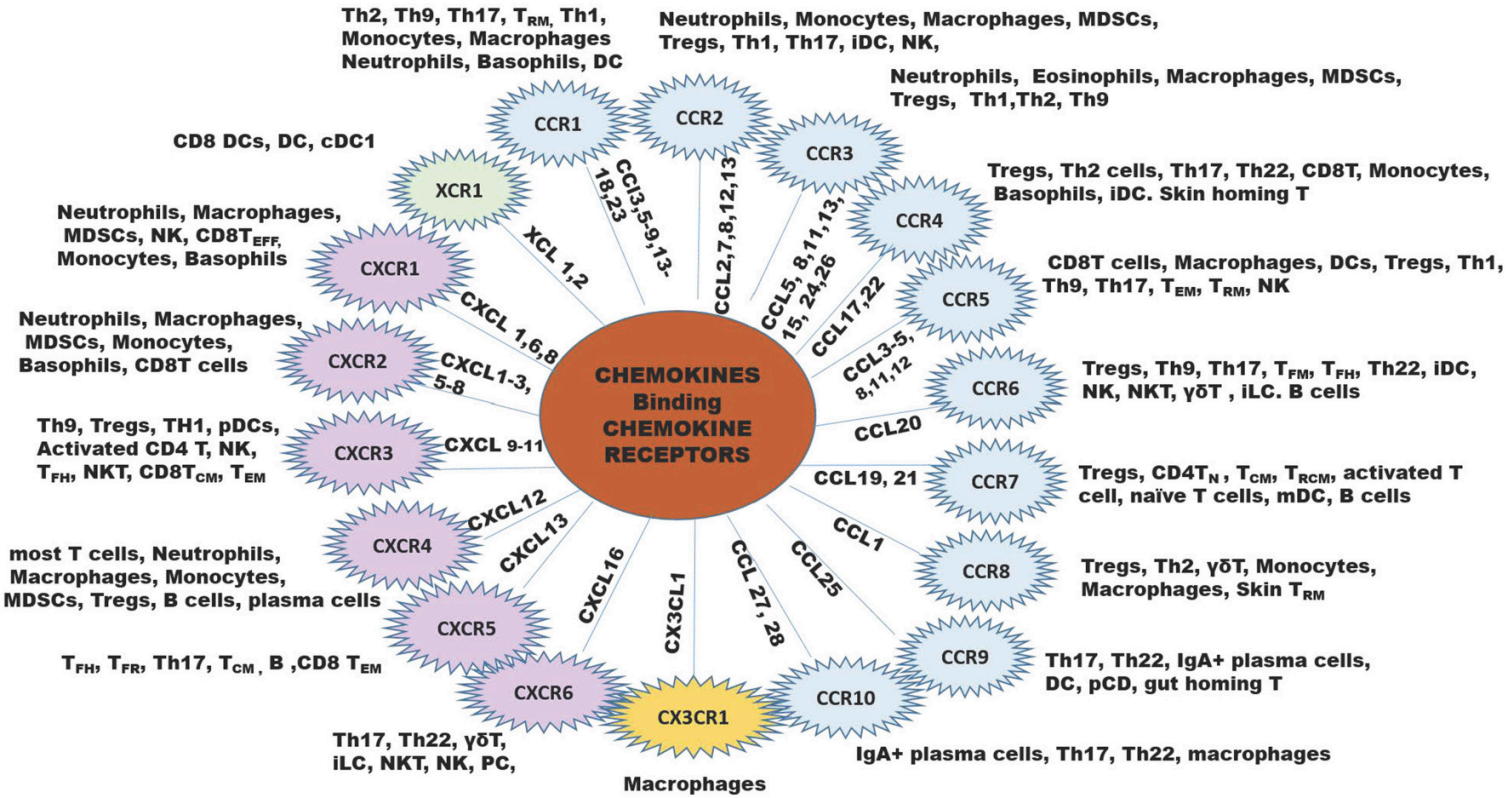
The chemokine family of chemokine ligands and chemokine receptors. The XC chemokine receptor is in green, CC chemokine receptors are in blue B, CX3CR chemokine receptor is in yellow, and CXC chemokine receptors are in lilac. The inner lines leading to each chemokine receptor shows the ligands that bind to the receptor. Outside the chemokine receptor wheel shows the types of cells that express the receptor to respond to the ligands for each chemokine receptor. B, B cell; iDC, immature DC; NK, natural killer cell; NKT, natural killer T cell; MDSCs, myeloid-derived suppressor cells; pDC, plasmacytoid DC; Th, T helper cell; T_CM_, central memory T cell; T_EFF_, effector T cell; T_FH_, follicular helper T cell; T_FR_, follicular regulatory T cell; T_N_, naïve T cells, T_RCM_, recirculating memory T cell (11, 12).

Since chemokines and their receptors are highly promiscuous, with most chemokines binding multiple receptors, and receptors binding multiple chemokine ligands. One must consider this complexity in reference to functional significance of each chemokine or receptor in reference to cancers (Figure 1).

Of key importance in immune surveillance is the recruitment of dendritic cells (DCs), CD4+Th1 cells, and CD8+ T effector cells to the tumor microenvironment. Chemokine receptors CCR4, CCR5, CXCR3, CXCR4, CCR6, and CCR7 play a pivotal role in the regulation of T cell homing to inflammatory sites (13). T cells (αβ, γδ, T_FM_, T_FH_, Th22, Tregs, ILCs, NKT), NK cells, B cells and immature DCs (14–16) are recruited to the tumor by CCL20 interaction with CCR6. CCL19 and CCL21 recruit Tregs, CD4T helper, T_CM_, T_RCM_, activated T cells, monocyte-derived dendritic cells (mDC) and B cells to the TME through interaction with CCR7 (7, 17–20). Dendritic cells home to XCR1, CCL3, CCL4, CCL5, CCL20, and CCL25 in the TME or LN (21–23). When antigen-specific CD4 T cells interact with DC, CCL3, and CCL4 are released and this can guide CCR5-positive naïve CD8+T cells into tissues for activation (24). As such, secretion of ligands for these receptors (CCL4/5 for CCR5, and CXCL9/10/11 for CXCR3) at the site of inflammation is necessary for the initiation of a specific immune response (25).

In contrast, tumor-promoting leukocytes are comprised of macrophages expressing arginase, IL4, IL10, and IL13, as well as myeloid-derived suppressor cells (MDSCs), T regulatory cells (Tregs) and specific B cell subsets. Ligands for chemokine receptors CCR1, CCR2, CCR3, and CCR5, CCR8, CXCR1, CXCR2, and CXCR4 recruit macrophages to the TME (4, 26–39). Neutrophils and myeloid derived suppressor cells (MDSCs) are recruited to the tumor through ligands for CCR2, CCR3, CXCR1, CXCR2, and CXCR4. Tregs express the chemokine receptors CCR2, 3, 4, 6, 7, 8, 10, CXCR3, and CXCR4 (40–48). Because the same chemokines that recruit anti-tumor leukocytes can also recruit pro-tumor leukocytes (for example CCL19 and CCL21 recruit both Tregs, mDCs, and activated T cells), therapeutically targeting chemokines or chemokine receptors in cancer is complicated.

For naïve T cells to become activated, antigen presenting DCs migrate from the developing tumor to the lympth node where they present antigen to the T cells via the T cell receptor (TCR) and stimulate a process that leads to T cell activation. CD4 cells can be activated by antigen presenting cells (APCs) and mature into helper cells [T helper type I cells (Th1) or T helper type II cells (Th2)]. Th1 cells produce cytokines including interferon-γ (IFNγ), tumor necrosis factor-alpha (TNFα), while Th2 cells secrete IL-4, IL-5, IL-10, and IL-13. The cytokines produced by the DCs influence the differentiation of naïve helper T cells into either Th1 or Th2 cells. For example, if DCs secrete IL-12, the naïve helper T cells differentiate into Th1 cells. Th1 cells express CD40L on their plasma membrane and this ligand binds to CD40 expressed by the DC or other APC. Engagement of CD40 on the DCs or other APC primes them to a higher activation level resulting in elevated expression of class I MHC, B7 and co-stimulating molecules such as 4-1BBL. When CD8+ T cells come into contact with one of these highly activated DCs, its TCRs recognizes the peptides presented by the MHC Class I molecule on the DC/APC. This, in turn, leads to the activation of CD8 T cell upon binding of its TCR to the MHC presented peptide(12).The clone subsequently expands in response to IL-2 induced stimulation of cell proliferation. CD4 T cells are important for the survival and expansion of activated CD8 T cell clones and for the survival of memory CD8 T cells during recall expansion, but there is some priming in the absence of Major Histocompatibility Complex, Class II (MHCII) activation (49). Different subsets of T cells migrate in response to a variety of chemokines (12). For example CCR7 is expressed on all naïve CD4 T cells and it's ligand CCL21 is expressed by the endothelial cells of the high endothelial venules (HEV) which are specialized vessels that facilitate lymphocyte recruitment. CCL21 is presented by heparin sulfate into the luminal surface (49). CCL19 can also bind to CCR7 on CD4 cells and is thought to mediate survival of naïve T cells as they move into the LN (50). Once in the LN, naïve CD4 T cells search for APCs using a random walk along a fibroblastic reticular cell network (51) which expresses adhesion molecules in addition to ligands for CCR7, CCL19, and 21, as well as CXCL12, which binds CXCR4. To escape the LN, CCR7 gradually becomes down-regulated and the CD4 cells bind the sphingosine-1-phosphate receptor 1 (S1PR1) (52) and follow S1P signals into the lymphatic vesicles, other LNs, or the circulation. FOXO1 is a key transcription factor in CD4 T cells, as is KLF2. FOXO1 regulates expression of CD62L and CCR7, while KLF2 represses CXCR3, CCR3 and CXCR5 expression (53).

When CD4 T cells are activated, there is upregulation of CXCR3 and CXCR5, both or which are associated with differentiation into T_H1_ cells (54) and can be linked to Bcl6 and cell division, though the order is controversial (55, 56). TCR engagement, IL12, IL21, and IFNγ expression along with induction of T-bet are associated with escape from a plastic state into a definitive Th1 phenotype (57). The cells migrate from the T zone to the B-T zone interface usingCXCR5 and EB12 (58) to escape areas with high IL-2. In contrast, contact with an environment high in IL-2 will suppress T_FH_ differentiation.

CD4+T cells undergo priming by DCs and upregulate CXCR3 expression, then CXCR3 mediates the migration of CD4+T cells between different DC populations in the LN. These CD8α+DCs are producing CXCL10 in response to IFNγ stimulation. CXCL9, CXCL10, and CXCL11 are produced by many cell types including fibroblasts, leukocytes, and keratinocytes and all bind CXCR3, though the most potent ligand in humans for CXCR3 is CXCL11 (59). CXCR3 is essential for T cell recruitment into tumors and through the thymus (60, 61) and Th1 cells also produce IFNγ that induces additional production of CXCL9 and 10 to enhance the recruitment of cytotoxic CD8+ T cells into the tumor (62).

Th2 cells express CCR4 and this receptor responds to ligands CCL17 and CCL22. CCR4 expression is induced in response to IL-4 and CCR4 expressing Th2 cells may also produce IL-4 (63–65). In contrast, those Th2 cells that express CCR8 produce IL-5 (66). Another key population of CD4 cells is the CD4+ memory T cells that express CCR7 and CD62L. These cells produced IL-2 when there is restimulation (67).

**Figure 2:**
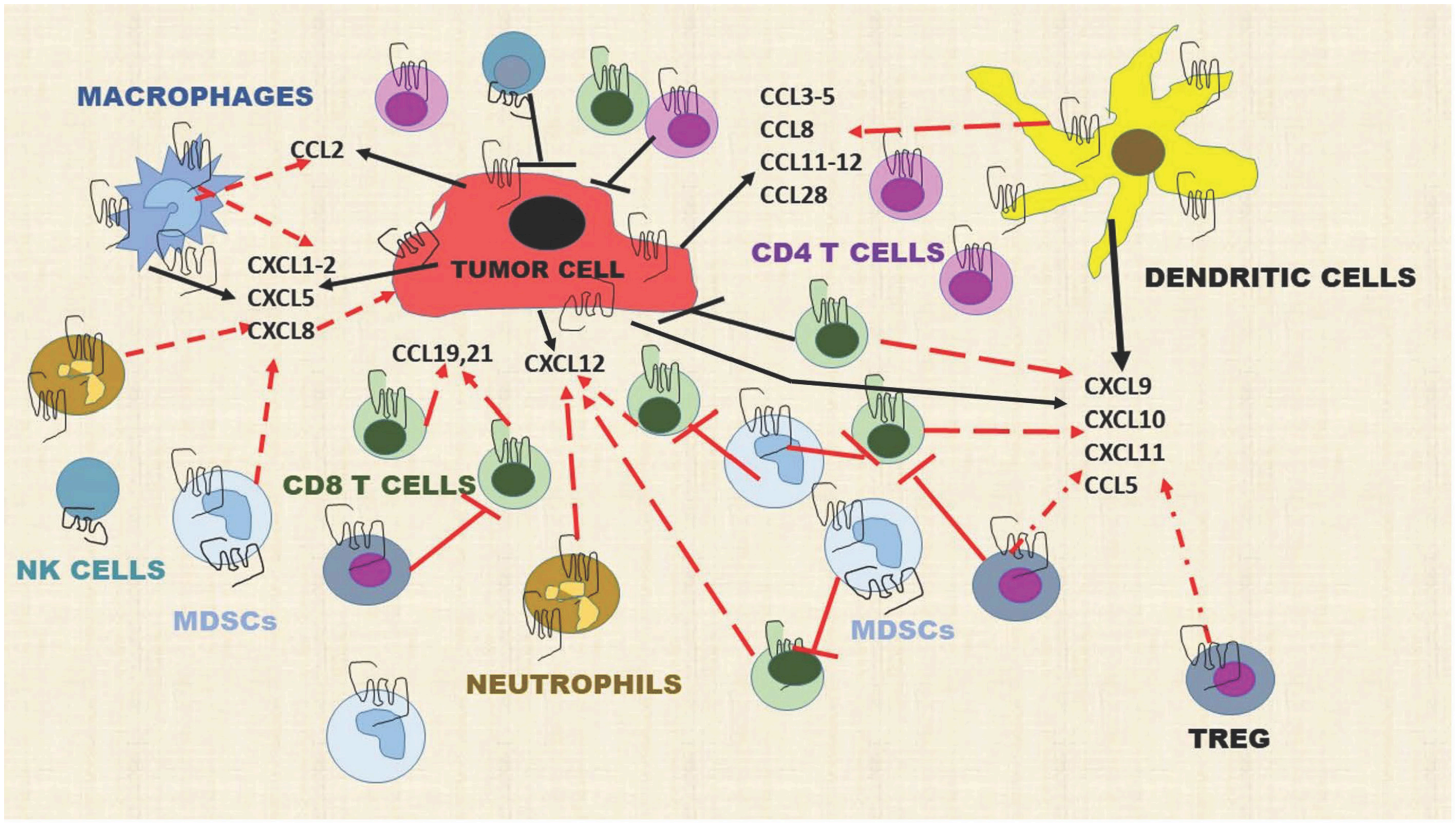
Chemokines produced by stromal cells, tumor cells, and immune cells dynamically modulate the immune landscape of the tumor microenvironment. Dashed red lines indicate a cell moving toward a chemokine gradient. Solid T red lines indicate inhibition. Solid black lines indicate chemokines released by a cell type. Solid black T lines indicate immune cell killing of tumor cells. This diagram includes representative chemokines recruiting immune cells but does not include all possible interactions.

In the tumor microenvironment, chemokines are produced by tumor cells, endothelial cells, mesenchymal stem cells (MSC), cancer-associated fibroblasts, myeloid cells, and neutrophils, providing a very rich “soil” to facilitate the recruitment of immune cells into the tumor microenvironment (TME). For example, tumor cells, macrophages, and neutrophils produce CXCL1, CXCL2, CXCL5, and CXCL8 and these chemokines recruit MDSCs, both the PMN-MDSCs and the Monocytic-MDSCs (68, 69). The MDSCs suppress the activity of CD8+T effector cells to prevent tumor cell killing by these cells. Dendritic cells (DCs), Tregs, CD8+ T cells, Th1, Th9, Th17, T_EM_, T_RM_, and macrophages are recruited into the TME by CCL3-5, CCL8, CCL11-12, and CCL28 (70). Mature DCs release CXCL5, CXCL9-11 and these chemokines recruit CD4+Th cells, CD8+T cells, Tregs, pDCs, NK, and NKT cells into the TME (71) (Figure 2). Additional interactions of chemokines and chemokine receptors that facilitate recruitment of diverse immune cells are shown in Figure 1.

## Tumor Chemokines and Patient Prognosis

**Figure 3:**
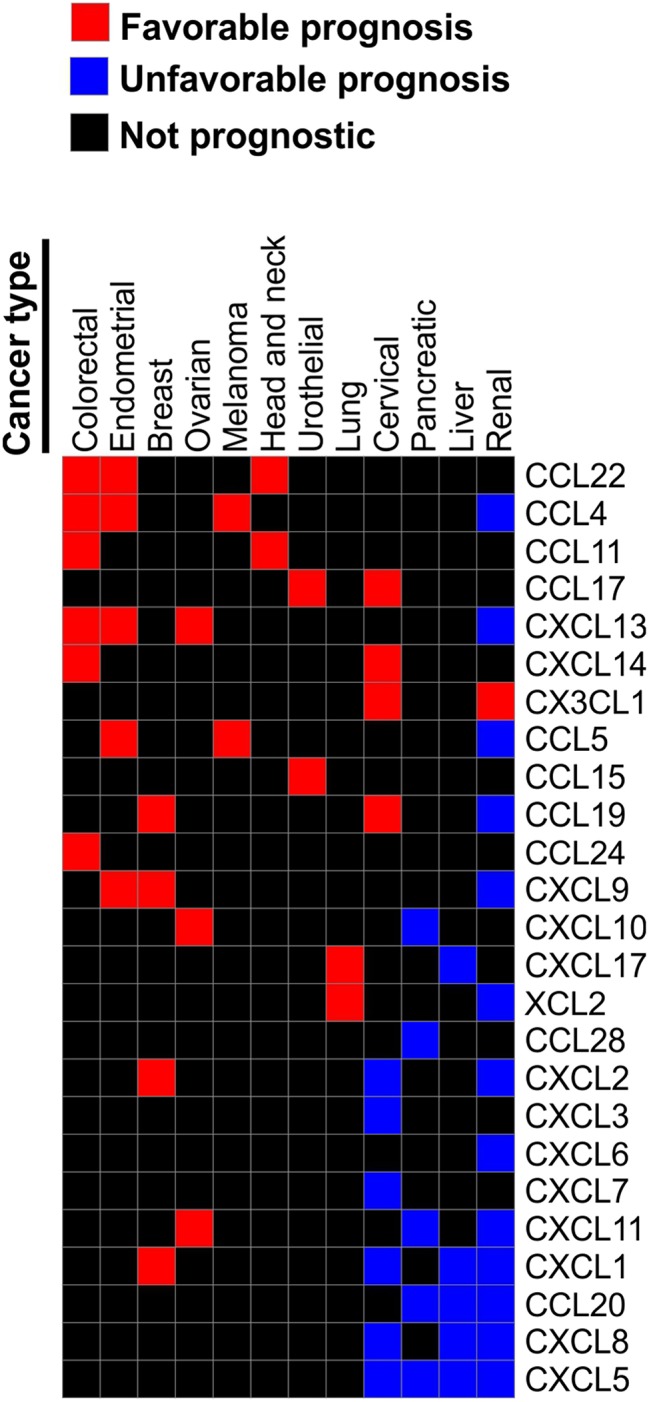
Chemokines associated with patient survival in various malignancies. Prognostic data was obtained from The Human Protein Atlas. We reviewed Kaplan-Meier plots for all cancers where high expression of indicated chemokine genes has significant (*p* < 0.001) association with patient survival. Based on this review we constructed a table where chemokines associated with better survival in one of the reviewed malignancies were assigned the value of “1.” Chemokines that were significantly associated with worse survival in a given malignancy were assigned the value of “−1.” Chemokines not strongly associated with survival (*p* > 0.001) were assigned the value of “0.” Chemokines that were not prognostic in any of the tested malignancies were excluded. Based on the resulting table the heat map was constructed using Morpheus online tool (https://software.broadinstitute.org/morpheus).

According to the analysis of the TCGA collection of human cancers using either The Human Protein Atlas (www.proteinatlas.org) (72, 73) or CBioPortal (74, 75), chemokine expression can be prognostic in many human cancers. However, same chemokines can be either favorable or unfavorable prognostic indicators depending on the type of malignancies. For instance, T cell-recruiting chemokines CXCL9, CXCL10, and CXCL11 are favorable prognostic indicators in ovarian cancer, but are unfavorable indicators for pancreatic and renal cancer. CXCL9 is also favorable in endometrial and breast cancer. Elevated expression of CXCL1 is unfavorable indicator in renal, liver and cervical cancers, but it is favorable in breast cancer. High CXCL5 is associated with poor survival in renal, liver, pancreatic and cervical cancer, while CXCL12 is not prognostic in any of the common TCGA malignancies. High expression of CCL4 and CCL5 are associated with better outcome in melanoma, endometrial, and colorectal cancer, but with worst outcome in renal cancer (Figure 3). Furthermore, a study of 14,492 distinct solid tumors (primaries and metastases) with at least 30 per tumor type revealed that a 12-chemokine expression signature (CCL2, CCL3, CCL4, CCL5, CCL8, CCL18, CCL19, CCL21, CXCL9, CXCL10, CXCL11, and CXCL13) correlated with the presence of tertiary lymph node-like structures and was also associated with better overall survival of the subset of melanoma patients (76). Moreover, loss of CCL5 expression was found to be associated with enhanced melanoma aggressiveness (77) and poor therapeutic response (78). Interestingly, tumor genomic instability can affect chemokine expression and patients' outcome. For instance, chromosomal instability in colorectal cancer can lead to deletion of the *CXCL13* gene which is associated with greater risk of tumor relapse (79). Of note, in human breast cancer CXCL13 is produced by follicular helper T cells which are linked with activation of adaptive antitumor humoral responses (80).

Thus, primary tumor data indicate that chemokines play an important role in tumor progression, which, in part, may relate to the direct effect of chemokines on cancer cell growth and metastasis (9). However, the main effect of chemokines is likely due to their ability to recruit specific subtypes of immune cells into the tumor that, in turn, can modulate tumor growth and metastasis. Indeed, immune cells within the tumor are among the key determinants of cancer outcome, based on the pan-cancer meta-analysis that correlated gene expression with overall survival outcomes in ~18,000 human tumors across 39 malignancies. This study showed that genes associated with immune cells, especially T cells, are the most significant indicators of favorable patient outcome (81). Furthermore, the presence of T cells or T cell expression signature within the tumor is associated with greater likelihood of response to immune checkpoint inhibitors (22, 76, 82–85). Below we summarize recent studies demonstrating that chemokine-mediated recruitment plays a central role in the regulation of the levels of different immune subtypes within the tumor.

## Chemokines Regulate Tumor Aggressiveness and Metastasis

### Pro-metastatic Chemokine Signaling in Tumor Cells

Tumor cells express a wide range of chemokine receptors, and there are extensive reports that tumor cells utilize both autocrine and paracrine pathways to respond to chemokines with altered migration, proliferation, and gene expression. Importantly, chemokine receptors have been reported to play a crucial role in maintenance of cancer stem cells. For example, a CXCR1 blockade has been shown to selectively target breast cancer stem cells (86) and its expression has been correlated with poor prognosis in breast cancer (87). CXCR1 and CXCR2 have been linked to melanoma tumor growth and metastasis (88–91).

Similarly, CCL2 expression by cancer-associated fibroblasts has been shown to support the growth of breast cancer stem cells (92), while CXCR4 was shown to be enriched in a subset of glioma cancer stem cells (93). Furthermore, CXCR2 is expressed in MSC and CXCR2 overexpressing MSCs can be used to accelerate mucosa wound healing (94). Both CXCR5 and CXCR4 are involved in metastasis of PCSLC prostate cancer stem-like cells (95), and inhibition of CXCR4 alters the homing of quiescent stem-like prostate cancer cells to bone (96). Furthermore, expression of the CXCR4 ligand, CXCL12, by tumor-associated fibroblasts has been shown to promote immune evasion in a murine model of pancreatic cancer, while targeting CXCR4 with specific antagonist AMD3100 facilitated immunotherapy response in these model (97). CCR5 has also been implicated in breast cancer growth and metastasis (98–100). These findings provide a rationale for targeting these chemokine receptors within the tumor microenvironment.

### Pro-metastatic Chemokine Signaling in Metastatic Niche

Chemokines play a crucial role in establishing the make-up of the “pre-metastatic niche.” Yang et al. reported that when CXCR2 and CXCR4 are inhibited, recruitment of MDSCs to the pre-metastatic niche of the lung is inhibited and, as a result, breast cancer metastasis to the lung is significantly reduced (37). Granot et al. reported that tumor-entrained neutrophils (TENs) inhibit metastatic seeding in the lungs by generating H_2_O_2_ and tumor-secreted CCL2 is a critical mediator of optimal anti-metastatic entrainment of G-CSF-stimulated neutrophils. Tumor entrained neutrophils inhibit seeding in the pre-metastatic lung (101). In contrast, Lavender reported that while *in vitro* delivery of CCL2 to 4T1 TENs enhanced the killing of the less aggressive 67NR variant of 4T1 tumor cells, intranasal delivery of CCL2 enhanced the seeding and outgrowth of tumor cells in the lung (102). However, it has been shown that patients with high CCL2 expressing basal-like, HER2+ and luminal B breast cancer exhibit a higher probability of longer survival in comparison to those patients with low expression of CCL2. These results are contradicted by findings showing that CCL2 and CCL3 are pro-tumor based upon their recruitment of pro-tumor macrophages into the TME (26). Presumably, the contribution of different chemokines to tumor growth and metastasis may be context dependent reflecting the overall complexity of cancer-associated chemokine-chemokine receptor network.

## Chemokines Facilitate “T Cell-Inflamed” Tumor Phenotype

Cytotoxic CD8 T cells are Th1-differentiated CD4 T cells are the main drivers of anti-tumor immunity, and there is a strong clinical and experimental evidence that chemokines are necessary to for the recruitment of these cells into the tumor. Analysis of patient samples indicates that chemokine expression is associated with T cell infiltrate. For example, in melanoma, the lack of CCR5 ligands (CCL3, CCL4, CCL5) and CXCR3 ligands (CXCL9 and CXCL10) has been associated with limited infiltration of antigen-specific T cells (103). The critical role of CXCR3 ligands in the recruitment of T cells into tumors of various origin has been well-documented (4). This critical role was further confirmed by the recent meta-analysis study which examined 5,953 cancer specimens from breast, colorectal, lung, ovary, melanoma, and head and neck carcinomas. This study demonstrated a positive correlation of CXCL9, CXCL10, and CXCL11 mRNA expression with the density of tumor-infiltrating T and NK cells (104). Interestingly, this study also uncovered a surprising negative correlation between the expression of CXCR3 ligands and neutrophil levels within tumors, indicating a possibility of a mutually exclusive pattern of T cell and neutrophil recruitment. Functional studies revealed that blockade of CXCL9 and CXCL10, or their receptor CXCR3, impairs recruitment of adoptively transferred T cells into melanoma tumors (61, 105). Furthermore, B16 melanoma tumors grow more rapidly in mice lacking CXCR3, and their tumors have lower levels of T cells as compared to wild-type mice. Notably, response to T cell-reactivating therapy, such as PD-1 blockade, is also impaired in CXCR3 knockout animals (105). These findings implicate CXCR3 ligands as major regulators of T cell tumor homing. Interestingly, there is evidence that tumors can find ways to neutralize anti-tumor chemokines within the tumor microenvironment. For example, a study Barreira da Silva et al. showed that dipeptidylpeptidase DPP4 produced by stromal cells within the tumor truncated and inactivated chemokine CXCL10 in transplanted murine melanoma tumors, resulting in reduced T cell infiltration and enhanced tumor growth and metastasis (106). These findings suggest that DPP4 inhibitors which are used as anti-diabetic drugs could potentially be used to stimulate tumor immunity. Indeed, the prospective clinical study showed that DPP4 inhibition can preserve the bioactive form of CXCL10 in humans (107) and a clinical trial of DPP4 inhibitor linagliptin with PD-L1-antagonist is underway (NCT03281369).

Certain C-C chemokines can also contribute to T cell recruitment into the tumor. Clinical data indicate that CCR5 ligands, CCL4, and CCL5, can promote anti-melanoma immune response. This observation is based on our analysis of the TCGA set of 287 melanoma samples which identified a robust association of the CD8+ T cell marker CD8A with the expression of chemokine CCL5 (78). One of the receptors for CCL5, CCR5, is expressed on T cells, and it has been reported to direct CD8 trafficking to sites of inflammation (24). However, mouse studies showed that CCR5 is dispensable for homing of T cells into melanoma (61). Recent studies indicate the critical role of CCL4 and CCL5 within the tumor microenvironment is the recruitment of cells of myeloid lineage that support adaptive anti-tumor T cell responses, such as dendritic cells. For instance, NK cell-derived CCL5 in cooperation with XCL1 has been shown to drive DC1 recruitment into the tumor (108). Furthermore, tumor-derived CCL4 has also been linked with the recruitment of DC cells in a mouse model of melanoma. These DCs, in turn, recruited cytotoxic T cells into the tumor by producing CXCR3 ligands CXCL9 and CXCL10 (109). Similar data were obtained in urothelial bladder cancer (110).

Besides CXCR3 and CCR5 ligands, additional chemokines are now emerging as key players in the regulation of anti-tumor immunity. For example, CXCL16 has been implicated in driving immune response against liver cancer by recruiting anti-tumor NKT cells. Sinusoidal endothelial cells were the major source of CXCL16 which was induced by gut microbiome-mediated primary-to-secondary bile acid conversion (111). Cremonesi et al. demonstrated that recruitment of T cells into colorectal tumors is controlled by many chemokines, including CCL5, CXCL9 and CXCL10, CCL17, CCL22, CXCL12, CXCL13, CCL20, and CCL17 (112). Expression of these chemokines was induced upon exposure of patient-derived colorectal cancer cells to gut microbiota and thus was sensitive to antibiotic treatment. These chemokines predominantly induced recruitment of T cells with an anti-tumor activity which was associated with improved survival in an animal model and clinical samples (112).

These reports suggest that many different chemokines contribute to anti-tumoral T cell recruitment. However, experimental evidence suggests that not all of these chemokines directly regulate T cell chemotaxis. For instance, an *in vivo* analysis of anti-tumoral T cell chemotaxis using competitive homing assay showed that key tumor-derived chemotactic factors are CXCR3 ligands, while CCL5, which was also produced by melanoma tumors, is dispensable for direct homing of T cells into the tumor (61). Furthermore, as shown by Yagawa et al. who used a standardized chemokine assay to test immune cell recruitment by 48 recombinant chemokines, resting CD4+ and CD8+ T cells displayed concentration-dependent chemo-attraction toward CCL19, CCL21, CXCL10, and CXCL12 and, to a lesser extent, toward CCL13, CCL16, CXCL9, CXCL11, CXCL13, and/or CXCL16 (113). None of the other tested chemokine molecules, including CCL4 and CCL5, were chemotactic for T cells in this experimental setting. These data suggest that the observed correlation of T cell markers and CCL5 observed in human melanoma tumors could be a result of indirect promotion of T cell recruitment or proliferation by myeloid and antigen-presenting cells recruited by CCL4 and CCL5. Notably, some chemokines may even play a role in repelling T cells as shown by Li et al. who identified CXCL1 as a determinant of the non-T-cell-inflamed microenvironment (114). In summary, these data point out that complex chemokine profiles orchestrate diverse immune microenvironment of tumors, including “T cell-inflamed” phenotype.

## Chemokines and Tumor Response to Immunotherapy

Analysis of samples from melanoma patients undergoing various immunotherapeutic treatments, including cancer vaccines and immune checkpoint blockade with CTLA-4 and PD-1 antagonists, revealed that tumors responsive to immunotherapy tend to be infiltrated with T cells, which is described as “T cell-inflamed” tumor microenvironment (22, 82–84). It is not yet fully understood why immune cells are present in some but absent in other tumors. It has been hypothesized that tumors with high mutation burden are more immunogenic because peptides derived from mutated proteins can serve as neo-antigens when bound by MHC molecules for presentation to T cells and thus can trigger an immune response (115, 116). However, a study of a TCGA tumor sample collection found no correlation between the T cell gene expression signature and mutational burden in any cancer type (117). An explanation of this interesting data came from the recent study by Cristescu et al. which analyzed over 300 patient samples across 22 tumor types from four KEYNOTE clinical trials (85). This study found that tumor mutational burden and a T cell-inflamed gene expression profile were independently predictive of response to the PD-1 antibody pembrolizumab. Notably, these parameters demonstrated a low correlation between each other, suggesting that they reflect distinct features of tumors that independently promote immunotherapy response. Consistent with this conclusion, tumors that exhibited both high mutation burden and prominent T cell signature were most likely to respond to PD-1 blockade (27% response rates). Tumors exhibiting only one of these immunotherapy response-promoting phenotypes had an intermediate likelihood of response (11–12%), while response rates were low on “T cell cold” tumors with low mutation burden (0% response rate) (85). These data suggest that many tumors, including potentially immunogenic tumors with high mutation burden, find ways to exclude immune cells to escape immune-mediated destruction. Indeed, regardless of the mutational load and ability to produce neo-antigen peptides, if tumor antigen-specific T cells are not mobilized to infiltrate the tumor, the presence of mutations and neoantigens is not going to be sufficient to mount anti-tumor immunity.

Based on this logic, chemokines are likely to facilitate immunotherapy responses by bringing immune cells with anti-tumor activity into the tumor and, thus, counteracting T cell exclusion. The data from patients' samples supports this hypothesis. For example, Ayers et al. published a gene expression signature that accurately predicts response to PD-1 therapy in patients with HNSCC and gastric cancer (23). Notably, several chemokine genes including *CCL5, CXCL9, CXCL10*, and *CXCL11* were in this signature. Furthermore, a Genentech-sponsored study of therapeutic anti-PD-L1 antibody showed a significant positive correlation between therapeutic response and baseline CXCL9 levels in melanoma. This correlation, however, did not reach statistical significance in NSCLC or renal carcinoma tumors (118). Interestingly, the same study found that fractalkine CX3CL1 negatively correlates with anti-PD-L1 response in all tested indications. This is an unexpected finding because this chemokine is generally associated with T-cell infiltration.

It is important to mention that chemokines are essential not only for the response to PD-1/PD-L1 therapeutic targeting, but they are also implicated in response to other immunotherapeutic agents. For instance, functional mouse studies revealed the requirement of CXCR3 ligands for response to anti-TIM-3 immune checkpoint inhibitor when administered in combination with chemotherapeutic drug paclitaxel (119). Of course, not all chemokines play a beneficial role in immunotherapy outcome. It has been shown that high levels of chemokines CCL3, CCL4, and CXCL8 in pre-treatment tumor specimens were associated with worse patient overall survival after anti-CTLA4 and Carboplatin/paclitaxel treatment in melanoma (120).

The key question that remains is how the expression of immunotherapy response-promoting chemokines is induced in tumors? An interesting hypothesis came from a study by Topalian's group which found that chemokines CCL5 and CXCL1 were upregulated in PD-L1-positive melanoma tumors along with IFNγ and several IFNγ-regulated genes based on the analysis of 49 archived melanoma specimens that were either PD-L1 positive or negative (121). Notably, Topalian's group also showed that CCL5 and CXCL1 had no direct effect on PD-L1 expression *in vitro*. The rationale for this study relates to the fact that PD-L1 positive tumors are more likely to respond to anti-PD-L1 immunotherapy, even though PD-L1 is not a definitive predictor of response (118, 122). The connection between chemokines and IFNγ was later confirmed in HNSCC and gastric cancer where CCL5 and CXCL9-11 along with a number of IFNγ-regulated genes comprised an expression signature associated with response to PD-1 blockade (23). However, it is not entirely clear from these correlative studies whether IFNγ stimulates chemokine expression in tumors or whether chemokines recruit immune cells that produce IFNγ. Perhaps both mechanisms take place *in vivo*. On the one hand, chemokines such as CXCL9-11 have been shown to be induced by IFNγ *in vivo* (www.interferome.org) (123). On the other hand, chemokines orchestrate tumor homing of cells that are the major producers of IFNγ, such as Th1-polarized CD4+ T, CD8+ T cells, and NK cells (124). IFNγ released by these cells activates JAK-STAT signaling in tumor and other cells of the tumor microenvironment which leads to increased PD-L1 surface display (125–128). This compensatory PD-L1 induction mediated by IFNγ inhibits the anti-tumor activity of T cells which is a key mechanism of adaptive immune resistance. Furthermore, Benci et al. showed that prolonged IFNγ signaling contributes to tumor growth as a result of expression of interferon-driven inhibitor ligands (IDILS) which, in addition to PD-L1, include TNF Receptor Superfamily Member 14/Herpes Virus Entry Mediator (TNFRSF14), galectin-9 (LGALS9), MHCII, CD28 Antigen Ligand 2/B7-2 (CD86), and the Interferon Stimulated genes (ISGs), such as Interferon-Induced Protein with Tetratricopeptide Repeats 1 (IFIT1) and MX Dynamin Like GTPase1 (MX1)(129). This same study showed that CRISPR ablation of multiple of these IDILS or ISGs enhances response to anti-CTLA4+anti-PD1 (129). This CRISPR ablation worked better than the addition of anti-LAG3 and or anti-TIM3. These data are complicated by reports of JAK1 mutation being associated with resistance to anti-PD1 (130).

In addition to driving adaptive immune resistance, IFNγ also promotes chemokine expression which, in turn, can recruit additional immune cells into the tumor (123). Based on these findings, a model can be proposed where IFNγ-producing immune cells increase tumor chemokines to recruit more immune cells that will further induce chemokine expression and so on. At the same time, tumor cells try to escape immune-mediated killing by inducing PD-L1 and other immune checkpoint proteins. The remaining question not explained by this model is how IFNγ-producing cells are recruited into the tumor in the first place. We and others have identified key molecular signals and pathways regulating basal chemokine expression in tumor cells that can be modulated therapeutically. We discuss these studies in the following chapter.

## Therapeutic Implications

### Chemokines as Therapeutic Targets

Accumulating evidence suggests that CXCR2 and CXCR4 are promising therapeutic targets in multiple malignancies. There are now over 2,400 publications describing a role for CXCR4 in cancer and over 300 publications describing a role for CXCR2 in cancer progression. These receptors are expressed on tumor cells, endothelial cells, leukocytes, including MDSCs. These studies provide significant evidence that CXCR2 and CXCR4 promote tumor growth through a variety of mechanisms (30, 37, 68, 131, 132). For example, Yang et al. demonstrated that targeted deletion of CXCR4 in myeloid cells reduced melanoma and breast cancer tumor growth through a mechanism that involved enhanced recruitment and activation of NK cells in the tumor. Likewise, systemic treatment with a CXCR4 antagonist also significantly inhibited tumor growth (131). Moreover, in an organotypic tumor spheroid-immune cell co-culture model inhibition of CXCL12 enhanced T cell recruitment and the anti-PD-1 immunotherapy response in a colon carcinoma cell model (133). Other reports show that ablation of CXCR2 signaling inhibited metastasis of in pancreatic adenocarcinoma in mouse models (114, 134–139) and improved response to anti-PD1 (114, 135, 140, 141). CXCR2 antagonism also inhibits metastasis of breast cancer, lung, ovarian, melanoma cells in mouse models (32, 33, 89–91, 142–148). A meta-analysis study of 2,461 patients revealed that CXCR2 predicts poor overall and relapse-free survival in laryngeal SCC, lung cancer, pancreatic ductal carcinoma, clear-cell renal cell carcinoma, and hepatocellular carcinoma, but not for digestive tract cancer (149).

Currently, clinical trials are ongoing with both CXCR2 and CXCR4 antagonist (150–153).

### Therapeutic Induction of Chemokines

Chemokines control infiltration of diverse immune cells into the tumors. The immune cell infiltrate, in turn, is essential for mounting an effective anti-tumor immune response with immunotherapy. Thus, therapies that induce chemokine secretion in tumors and restore immune cell entry into non-inflamed tumors are likely to facilitate immunotherapy response. One of the previously explored approaches to induce infiltration of T cell into the tumors was to inject them directly with interferons. In a mouse model interferon injection into melanoma tumor-induced chemokine production and improved response to anti-PD-L1 therapy (154). One drawback of this approach is that since not all melanoma lesions are injectable, this strategy may miss potential micrometastases and therapeutic effects are likely to be transient. Indeed, a recent clinical study in melanoma patients did not find increased T cell infiltration after a single intra-tumoral injection of IFNγ (34). Other studies reported that chemo-and radio-sensitivity could increase chemokine expression (155, 156). However, melanoma tumors are notoriously resistant to chemotherapy and radiation.

We have discovered that senescent-inducing drugs increase chemokine secretion by melanoma cells (78). Senescence is a cell state of irreversible (or stable) cell cycle arrest accompanied by an induction of a complex secretory program known as senescence-associated secretory phenotype (SASP) (157). Using small molecules targeting cell cycle kinases, such as alisertib that inhibits mitotic kinase Aurora A, or palbociclib that inhibits CDK4/6, to induce senescence we demonstrated that the melanoma SASP includes a number of chemokines implicated in T cell trafficking (78, 158, 159). These chemokines included CCL5 and CXCR3 ligands which are up-regulated in tumors responsive to PD-1-targeting immune checkpoint therapy (23). Taken together, these data suggest that senescence-inducing therapy promotes chemokine secretion in melanoma cells which facilitates an inflamed tumor microenvironment.

Another approach to re-activate chemokine expression in immunologically cold tumors is by targeting the epigenetic blocks that impede chemokine expression in tumor cells. For instance, treatment of ovarian cancer cells with epigenetic modifiers reversed the EZH2 and DNMT1 suppression of expression of the CXCR3 ligands, CXCL9, and CXCL10, resulting in T cell influx into the tumor and improved response to T cell transfer and anti-PD-L1 blockade therapy (160). Interestingly, another study showed that DNMT1 inhibitor treatment induced expression of CXCL12 in osteosarcoma tumors. Activation of CXCR4 by CXCL12 has been reported to have pro-tumor activity. In contrast, in the context of DNMT1 inhibition in osteosarcoma, activation of the CXCL12-CXCR4 axis reduced metastasis and promoted T cell recruitment (161). Expression of CCL5 can also be epigenetically regulated as shown by the study in non-small cell lung cancer showing that a combination of DNA-demethylating agents with histone deacetylase inhibitors reversed tumor immune evasion and modulated the T cell phenotype away from a T cell exhaustion state toward memory and effector T cell phenotypes (162). These experiments indicate that epigenetic modifiers can be utilized for cancer treatment to rescue expression of key chemokines important for the recruitment of T cells and DCs to the tumor.

Also, viral delivery of chemokines can be used to increase T cell homing into the tumor and promote immunotherapy response. For instance, intra-tumoral injection of vaccinia virus delivering CXCL11 promoted response to adoptive T cell therapy and vaccines (163). In addition, it has been shown that oncolytic viruses can enhance secretion of CXCL2 and CXCL10 chemokines by tumors (164). Another promising approach to elevate chemokine levels within the tumor is nanoparticle delivery as demonstrated by CXCL10-loaded folate-modified chitosan nanoparticles that showed anti-tumor activity (165). Another study showed that resistance to PD-L1 blockade could be overcome by targeting tumors with tumor necrosis factor superfamily member, LIGHT. Administration of antibody-guided LIGHT activated lymphotoxin-beta receptor signaling which, in turn, facilitated production of chemokines CCL21 and CXCL13 that recruited T cells into the tumor (166). Finally, immune adjuvants, including double-stranded (ds) RNAs of Sendai Virus (SeV), poly-I:C, and rintatolimod (poly-I:C12U), has been shown to promote the production of CXCR3 ligand within the tumor (167). In glioblastoma poly(I:C) stimulated expression of chemokines CXCL9, CXCL10, CCL4, and CCL5 (167). Similarly, an engineered RIG-I agonist-induced expression of lymphocyte-recruiting chemokines in breast cancer cells (168). Altogether, these approaches of delivering agents that elevate levels of T cell-recruiting chemokines within the tumor can be used to stimulate anti-tumor immunity when tumors are in an injectable location.

## Concluding Remarks

In the last 30 years, we have made extensive progress in identifying chemokines and chemokine receptors, characterizing their roles in the development of the immune system, in angiogenesis, wound healing, inflammation, tumorigenesis, and host defense. Extensive effort was put into developing antagonists of chemokine receptors and some of these were investigated in various clinical trials. CCR5 antagonists, like maraviroc, have been developed and used in AIDs patients with some success (169). CXCR2 antagonists are currently in clinical trials to block MDSC recruitment to tumors and the pre-metastatic niche (NCT03177187 in metastatic castration-resistant prostate cancer (not yet recruiting). CXCR2 antagonists are also being evaluated in combination with immune checkpoint inhibitor pembrolizumab in advanced solid tumors (NCT03473925) and in metastatic melanoma (NCT03161431, not yet recruiting) (ClinicalTrials.org). CXCR4 antagonists have been and are in clinical trials: NCT02179970–to assess safety of continuous IV administration of plerixafor in patients with advanced pancreatic, ovarian and colorectal cancers (recruiting); NCT03277209–continuous IV administration of plerixafor to assess impact on immune microenvironment in patients with pancreatic, ovarian and colorectal adenocarcinomas (active but not recruiting); NCT02605460-chemo-sensitization before hematopoietic stem cell transplantation in patients with acute leukemia in complete remission–recruiting; NCT02737072–LY2510924 combined with durvalumab for solid tumors (terminated and results not posted); NCT01068301—a Phase I study plerixafor in combination with fludarabine, thiotepa, and melphalan for a second allogeneic stem cell transplantation has been completed but results are not posted; NCT01010880—safety study of CXCR4 antagonist in multiple myeloma patients-study was completed but no results are posted. Additional trials are ongoing for the CXCR4 antagonist BL-040 in NSCLC (NCT03337698), in AML in combination with atezolizumab (NCT03154827), in metastatic pancreatic cancer (NCT02907099), and in aplastic anemias or hypoplastic myelodysplastic Syndrome (NCT02462252) and several others. In addition, the Polyphor CXCR4 antagonist, balixafortide, combined with eribulin has completed Phase I trials in HER2-negative metastatic breast cancer patients and demonstrated an objective response in 16/54 evaluable patients (30%) with an additional 25 patients exhibiting stable disease (46%) (153). Xue et al. have recently reviewed additional reports showing CXCR4 is a potential target for cancer (170). Similarly, therapeutic approaches to increase chemokine expression in tumors to facilitate anti-tumor immune response are also explored in clinical studies. This includes trials of combined epigenetic and immunotherapy agents, such as DNA demethylating drug azacitidine with anti-PD-1 immunotherapeutic pembrolizumab (NCT03264404) or with anti-PD-L1 antibody avelumab (NCT03699384), as well as HDAC inhibitor entinostat and anti-PD-1 agent pembrolizumab (NCT02437136) and similar approaches (171). It will be interesting to follow the results from these ongoing clinical trials to learn what works and what revisions are needed to successfully modulate chemokines and chemokine receptors in combination with other key targets for treatment of cancers.

## Author Contributions

All authors listed have made a substantial, direct and intellectual contribution to the work, and approved it for publication.

### Conflict of Interest Statement

The authors declare that the research was conducted in the absence of any commercial or financial relationships that could be construed as a potential conflict of interest.
